# Combined Posterior and Interlobar Approaches Enable S10 Segmentectomy for a Centrally Located Intrapulmonary Schwannoma Diagnosed by Endobronchial Ultrasound-Guided Transbronchial Needle Aspiration: Surgical Strategy and Technical Consideration

**DOI:** 10.70352/scrj.cr.25-0501

**Published:** 2025-10-17

**Authors:** Tomonari Oki, Shuhei Iizuka, Hideki Miwa, Yoshiro Otsuki, Toru Nakamura

**Affiliations:** 1 Department of General Thoracic Surgery, Seirei Hamamatsu General Hospital, Hamamatsu, Shizuoka, Japan; 2 Department of Respiratory Medicine, Seirei Hamamatsu General Hospital, Hamamatsu, Shizuoka, Japan; 3 Department of Pathology, Seirei Hamamatsu General Hospital, Hamamatsu, Shizuoka, Japan

**Keywords:** intrapulmonary schwannoma, EBUS-TBNA, segmentectomy, benign pulmonary tumor

## Abstract

**INTRODUCTION:**

Intrapulmonary schwannoma is a rare benign tumor that often presents diagnostic and therapeutic challenges due to its nonspecific radiological findings and central location. Establishing a histological diagnosis before surgery is crucial to avoid unnecessary extensive lung resection.

**CASE PRESENTATION:**

A 46-year-old non-smoking woman presented with an incidental 2.4 cm pulmonary nodule centrally located in segment 10 (S10) of the left lower lobe. CT showed the tumor compressing the bronchus and pulmonary artery without direct airway communication, making transbronchial biopsy unfeasible. Endobronchial ultrasound-guided transbronchial needle aspiration (EBUS-TBNA) successfully obtained tissue samples, and histopathological examination revealed spindle cells with diffuse S100 protein positivity, confirming the diagnosis of an intrapulmonary schwannoma. Given the benign nature of the tumor, an S10 segmentectomy was planned. Preoperative 3D CT suggested that while the pulmonary vein (V10) could be approached posteriorly, the artery (A10) and bronchus (B10) would require an interlobar approach due to the tumor’s central location. Intraoperative findings confirmed this, and the tumor was successfully resected. The patient had an uneventful recovery and was discharged on POD 4. Final pathology confirmed the diagnosis of an intrapulmonary schwannoma and negative margin.

**CONCLUSIONS:**

Preoperative histological diagnosis of an intrapulmonary schwannoma by EBUS-TBNA is both feasible and clinically advantageous, enabling a limited anatomical resection. A preoperative strategy that incorporates both the tumor’s benign nature and its central location is essential for achieving optimal surgical outcomes.

## Abbreviations

EBUS-TBNA
: endobronchial ultrasound-guided transbronchial needle aspiration
FDG–PET
: fluorodeoxyglucose–positron emission tomography
PAVM
: pulmonary arteriovenous malformation
S10
: segment 10
TBB
: transbronchial biopsy

## INTRODUCTION

Intrapulmonary schwannoma is an exceptionally rare benign tumor originating from Schwann cells of the peribronchial autonomic nerves.^1–4)^ These tumors are typically located in the central pulmonary regions and usually demonstrate extraluminal growth without airway communication. Preoperative diagnosis is often challenging due to their radiological features mimicking low-grade malignancies such as carcinoid tumors and their limited accessibility via bronchoscopy.^1,4–6)^ Although a limited resection is generally sufficient to achieve a cure given its benign nature, its frequent central location may require more extensive lung resection in the absence of a histological diagnosis.^1,3,4)^

Herein, we report the case of a centrally located intrapulmonary schwannoma diagnosed preoperatively using endobronchial ultrasound-guided transbronchial needle aspiration (EBUS-TBNA), which enabled a limited resection. We also describe the surgical strategy and technical considerations unique to this case.

## CASE PRESENTATION

**Fig. 1:**
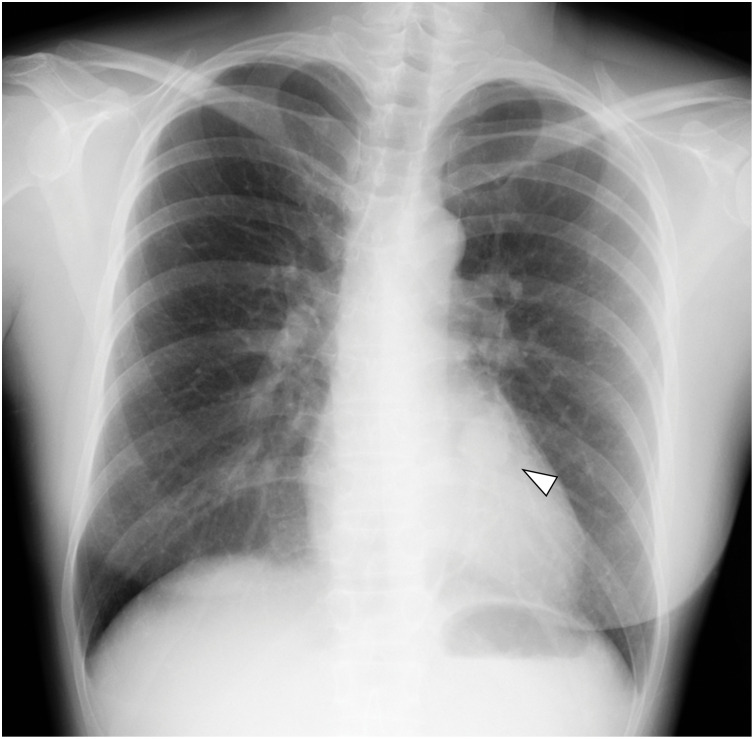
Preoperative chest radiograph showing the tumor (white arrowhead).

**Fig. 2:**
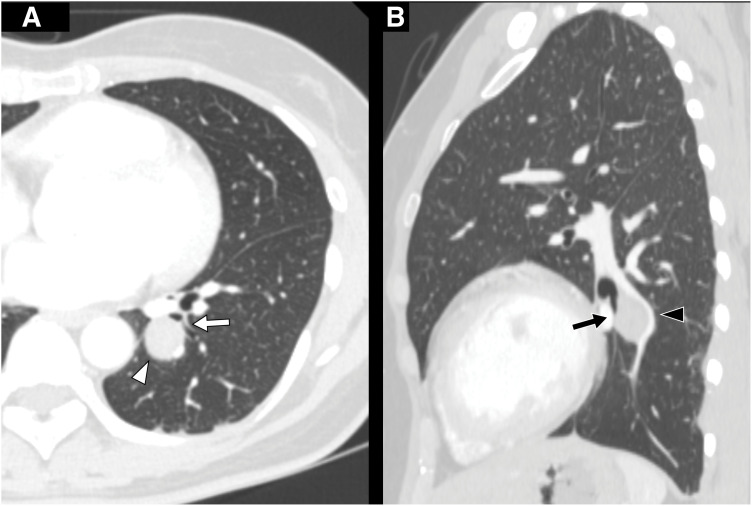
Chest CT images. (**A**) Axial CT showing a well-circumscribed 2.4 cm nodule in left S10 (white arrowhead), adjacent to the central portion of bronchus B10 (white arrow). (**B**) Sagittal CT demonstrating compression of pulmonary artery A10 (black arrowhead) and stenosis of bronchus B10 (black arrow) caused by the tumor. S10, segment 10

**Fig. 3:**
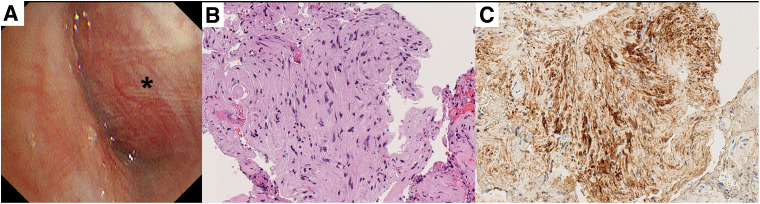
Bronchoscopy and histopathological findings. (**A**) Bronchoscopy showing extrinsic compression of B10. EBUS-TBNA was performed with the bronchoscope pressed against the bronchial wall at the asterisk. (**B**) Hematoxylin and eosin staining (× 20) showing spindle cell proliferation with minimal atypia. (**C**) Immunohistochemistry (× 20) confirming diffuse S100 protein positivity. EBUS-TBNA, endobronchial ultrasound-guided transbronchial needle aspiration

A 46-year-old non-smoking woman with no history of malignancy was referred to our hospital following the incidental detection of an abnormal shadow on chest radiography (**Fig. 1**). Chest CT revealed a well-circumscribed 2.4 cm nodule located in segment 10 (S10) of the left lower lobe (**Fig. 2A**). The pulmonary artery (A10) and the bronchus (B10) were compressed by the tumor (**Fig. 2B**). Differential diagnoses based on CT included pulmonary carcinoid, sclerosing pneumocytoma, and pulmonary hamartoma. Bronchoscopy revealed extrinsic compression of B10 (**Fig. 3A**). The subsegmental branches distal to the stenosis could not be visualized because of tumor-induced narrowing, but no epithelial irregularities were observed. As there was no bronchus leading directly into the tumor (**Fig. 2A**), transbronchial biopsy (TBB) was deemed unfeasible. Therefore, EBUS-TBNA was performed. On EBUS examination, a well-defined hypoechoic lesion was visualized, and 4 needle passes were performed to obtain samples. Rapid on-site cytologic evaluation was negative. The procedure was conducted using a BF-UC290F bronchoscope (Olympus, Tokyo, Japan) and NA-U401SX-4021 aspiration needle (Olympus). Histopathological examination revealed spindle cell proliferation with minimal atypia (**Fig. 3B**). Immunohistochemistry revealed diffuse S100 protein positivity, confirming diagnosis of an intrapulmonary schwannoma (**Fig. 3C**). No additional cellular components or malignant features such as necrosis, marked nuclear atypia, or mitotic activity were identified, thereby excluding neurofibroma and malignant peripheral nerve sheath tumor.

**Fig. 4:**
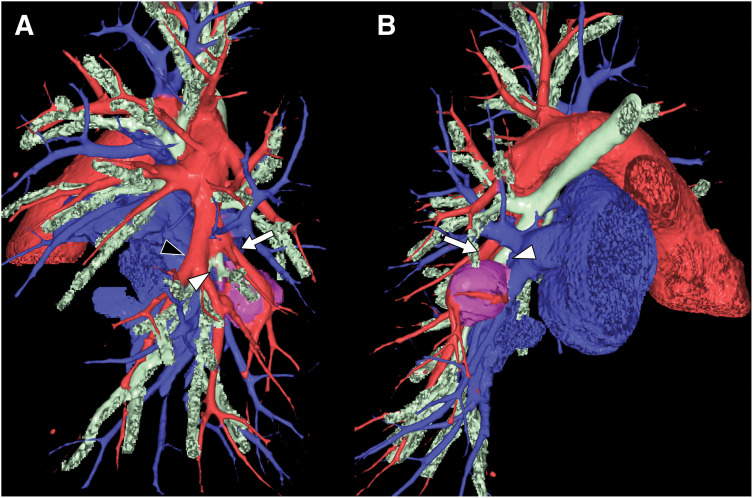
Preoperative 3D-CT. (**A**) Interlobar view: A10 (white arrow) is clearly visible and the bifurcation of B10 (white arrowhead) can be exposed after transection of A10 and retraction of A8+9 (black arrowhead). (**B**) Posterior view: Due to the tumor location, A10 (white arrow) and the B10 bifurcation (white arrowhead) are not visible.

Because the tumor narrowed B10 and posed a risk of atelectasis or pneumonia in S10, surgical resection was indicated. Given its central location surrounded by the pulmonary artery, pulmonary vein, and bronchus, wedge resection or enucleation was anatomically unfeasible. Considering the benign pathology, we opted for a limited resection without a wide margin, despite its central location within S10. A posterior basal segmentectomy (S10 segmentectomy) was planned. Preoperative 3D-CT revealed that the pulmonary vein (V10) could be accessed from the posterior hilum (**Fig. 4A**). By contrast, A10 and B10, situated ventral to the tumor, were unlikely to be accessible and were anticipated to require exposure via the interlobar fissure (**Fig. 4B**). Furthermore, because the tumor was located centrally in B10, stapler transection was not feasible, and hand-sewn closure of the bronchial stump was anticipated. In addition, the proximal bronchial stump was expected to retract deeply into the lung parenchyma after transection, making suturing impossible posteriorly; therefore, an interlobar approach was also required. Accordingly, a combined posterior and interlobar approach was planned.

**Fig. 5:**
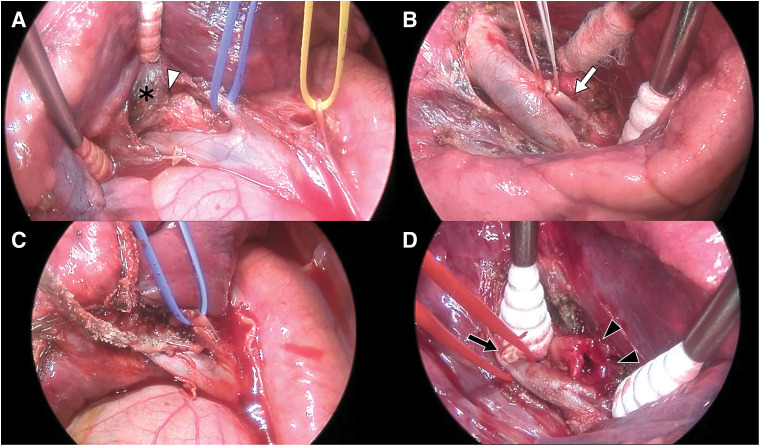
Intraoperative findings. (**A**) Posterior approach: The tumor (*) was adherent to B10 and lung parenchyma, preventing exposure of A10 ventral to B10. Normally, A10 can be easily identified at the site indicated by the white arrowhead. (**B**) Interlobar approach: A10 (white arrow) was readily exposed and safely divided. (**C**) Posterior view after the tumor resection: The proximal bronchial stump of B10 was retracted into the lung parenchyma and was not identified. (**D**) Interlobar view after the tumor resection: The bronchial stump of B10 (black arrowheads) is clearly visible following the division of A10 (black arrow) and retraction of A8+9.

Surgery was performed via a mini-thoracotomy with thoracoscopic assistance. An 8-cm incision was made at the 5th intercostal space along the posterior axillary line, and a 1.2-cm thoracoscopic port was placed at the 8th intercostal space along the mid-axillary line. The procedure was initiated with a posterior approach, and V10 was ligated with 2-0 silk suture and divided using an ultrasonically activated device. Following the dissection of intersegmental veins V6 and V8+9, the tumor was identified dorsally. However, the tumor, located at the proximal B10, was firmly adherent to the adjacent parenchyma, and the bifurcation of B10 could not be identified posteriorly. Moreover, A10, which lies ventral to B10, could not be exposed (**Fig. 5A**). As predicted, dissection of B10 and A10 was difficult via the posterior approach, and the operative strategy was promptly converted to an interlobar approach. After switching to an interlobar approach, A10 was safely dissected, doubly ligated with 2-0 silk suture (**Fig. 5B**), and divided. Retraction of A8+9 facilitated adequate exposure of B10. Because of the tumor’s central location, bronchial transection with a stapler was not feasible. B10 was therefore transected with a scalpel approximately 5 mm proximal to the tumor. The intersegmental plane was divided with a stapler, and the S10 segment was successfully resected. As anticipated, the proximal bronchial stump could not be visualized from the posterior approach (**Fig. 5C**); however, a clear surgical view was obtained interlobarly (**Fig. 5D**). The bronchial stump was closed using hand-sewn sutures with 3 interrupted 4-0 nonabsorbable monofilament sutures under direct visualization.

**Fig. 6:**
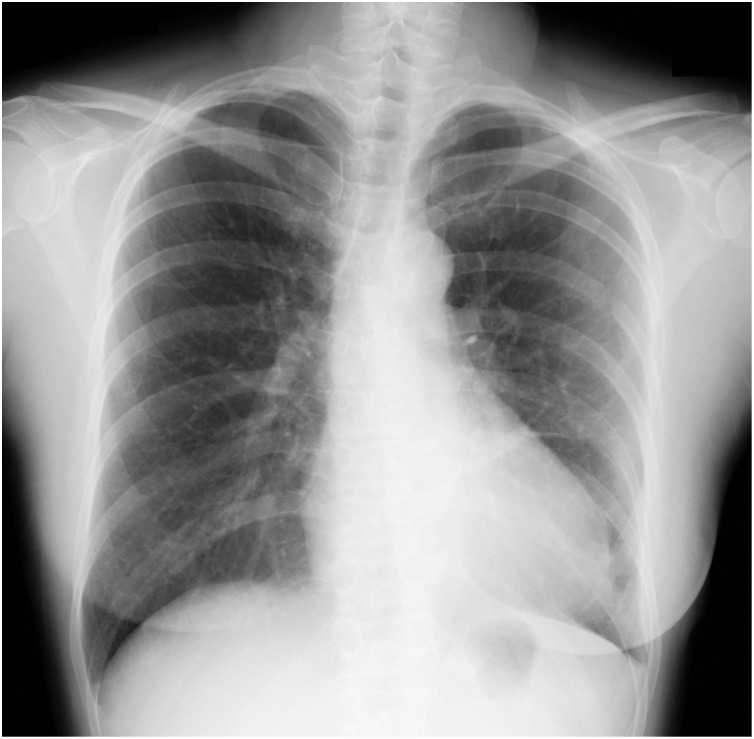
Chest radiograph at 3 months postoperatively demonstrating full lung re-expansion.

The patient’s postoperative course was uneventful. The chest drain was removed on POD 3, and the patient was discharged on day 4. Follow-up radiographs demonstrated adequate lung re-expansion (**Fig. 6**), and she resumed normal activities within 1 month.

**Fig. 7:**
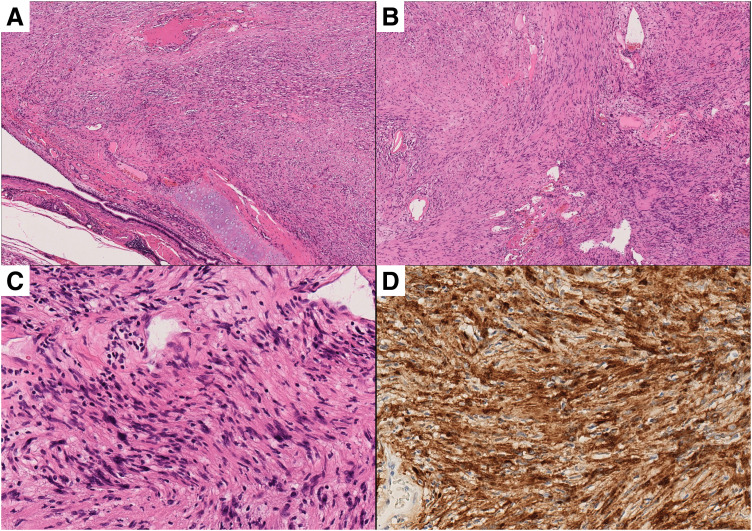
Histopathological features. (**A**) Hematoxylin and eosin (× 4): Tumor compressing the bronchus without intervening tissue. (**B**) Low-power view (× 4): Spindle cell proliferation with alternating hyper- and hypocellular areas. (**C**) High-power view (× 20): Fascicular growth pattern. (**D**) Immunohistochemistry (× 20): Diffuse S100 positivity.

Histopathological examination revealed tumor compression of the bronchial wall without intervening tissue (**Fig. 7A**). The tumor consisted of spindle cells arranged in alternating hyper- and hypocellular areas (**Fig. 7B**), displaying a fascicular pattern (**Fig. 7C**). Immunohistochemistry reconfirmed diffuse S100 positivity, consistent with an intrapulmonary schwannoma (**Fig. 7D**), and the bronchial resection margin was free of tumor.

## DISCUSSION

Intrapulmonary schwannoma is exceedingly rare, comprising approximately 0.2% of all pulmonary neoplasms.^2)^ Most thoracic schwannomas originate from sympathetic, intercostal, or vagus nerves within the chest wall or posterior mediastinum. By contrast, intrapulmonary schwannomas that arise from peribronchial autonomic nerves, are often centrally located, and tend to grow extraluminally, thus lacking airway communication.^1,3,4)^ Although a complete resection with a minimal margin is curative and associated with an excellent prognosis, their central location often necessitates a lobectomy or even bronchoplasty in the absence of preoperative diagnosis.^1,3,4)^

Radiologically, these tumors typically appear as well-circumscribed nodules on CT with low-to-moderate uptake on fluorodeoxyglucose–positron emission tomography (FDG–PET), mimicking low-grade malignancies such as carcinoid tumors.^6)^

Although histological confirmation is essential for a limited resection, it is often challenging due to the absence of airway communication. As TBB relies on the presence of a bronchus leading to the tumor, its diagnostic yield is limited in cases of extrinsic compression without airway involvement.^7)^ To our knowledge, there have been no reports of a preoperative diagnosis of intrapulmonary schwannoma using TBB. By contrast, EBUS-TBNA enables sampling of peribronchial lesions regardless of bronchial communication^7,8)^ and has been reported to be diagnostic in intrapulmonary schwannoma,^9)^ including the present case. While no established diagnostic algorithm exists due to the rarity of this tumor, EBUS-TBNA appears to be a promising tool.

A critical safety consideration is the exclusion of pulmonary arteriovenous malformation (PAVM) prior to EBUS-TBNA using contrast-enhanced CT. PAVM, like schwannoma, may appear as a sharply defined pulmonary nodule.^10)^ Biopsy of an unrecognized PAVM can result in life-threatening hemorrhage or paradoxical embolism.^11)^ By contrast, no severe complications such as bleeding have been reported following EBUS-TBNA for intrapulmonary schwannoma.^9,12)^

Surgical strategy should be tailored to balance the tumor’s benign nature and central location. Similar to pulmonary hamartomas, which are a representative benign tumor of the lung, schwannomas are generally not associated with metastasis or significant local invasion.^13)^ In the case of pulmonary hamartomas, which are typically well-encapsulated and located within the lung parenchyma, enucleation through a parenchymal incision and direct tumor exposure is considered an appropriate surgical approach. Numerous reports have demonstrated favorable long-term outcomes with this technique.^14)^ By contrast, schwannomas originate from peripheral nerves and are often continuous with adjacent nerve fibers. As such, direct manipulation or exposure of the tumor during surgery may increase the risk of capsule disruption and exposure of tumor contents. Indeed, there has been a reported case of a port site recurrence following video-assisted thoracoscopic resection of a chest wall schwannoma, in which the tumor was extracted without the use of a retrieval bag. This suggests that, despite their benign nature, schwannomas may still pose a risk of local tumor implantation if handled improperly.^15)^

Therefore, while a radical resection with a wide surgical margin and lymph node dissection, as performed for lung cancer, are not necessary for schwannomas, enucleation involving direct tumor exposure, as is standard in hamartoma resection, should be approached with caution. To minimize the risk of intraoperative implantation, complete tumor removal with minimal but sufficient surgical margins, while preserving the integrity of the tumor capsule, is advisable; thus anatomical segmentectomy was considered the most appropriate for this case.

Given its central location in S10, adjacent to both S6 and S9, the present case would have necessitated a left lower lobectomy if suspicion of malignancy had not been excluded. Thanks to the preoperative histological diagnosis by EBUS-TBNA, segmentectomy was justified.

S10 segmentectomy is technically demanding due to the deep and complex anatomy of the hilar structures involved.^16,17)^ Recently, the posterior approach for S10 segmentectomy has become increasingly favored, as it allows dorsal access to V10, B10, and A10. However, in our case, preoperative imaging revealed that A10 and B10 were inaccessible dorsally due to the tumor’s central location, thereby necessitating an additional interlobar approach. Moreover, bronchial suturing was anticipated and considered feasible via the interlobar approach. By combining posterior and interlobar approaches, rather than relying solely on the posterior route, we achieved a safer and more feasible operative strategy tailored to the tumor’s central location. Careful preoperative planning facilitated intraoperative decision-making and enabled an uneventful surgical procedure.

## CONCLUSIONS

Preoperative histological diagnosis of intrapulmonary schwannoma by EBUS-TBNA is both feasible and clinically advantageous, enabling limited anatomical resection. A preoperative strategy that incorporates both the tumor’s benign nature and its central location is essential for achieving optimal surgical outcomes in the management of this rare pulmonary tumor.

## DECLARATIONS

### Availability of data and materials

Data sharing is not applicable to this article as no datasets were generated or analyzed during the current study.
